# Salt adaptability in a halophytic soybean (*Glycine soja*) involves photosystems coordination

**DOI:** 10.1186/s12870-020-02371-x

**Published:** 2020-04-10

**Authors:** Kun Yan, Wenjun He, Lanxing Bian, Zishan Zhang, Xiaoli Tang, Mengxin An, Lixia Li, Guangxuan Han

**Affiliations:** 1 CAS Key Laboratory of Coastal Environmental Processes and Ecological Remediation, Yantai Institute of Coastal Zone Research (YIC), Chinese Academy of Sciences(CAS), Shandong Key Laboratory of Coastal Environmental Processes, YICCAS, Yantai, Shandong 264003 P. R. China; 2 College of Life Sciences, Yantai University, Yantai, 264005 P. R. China; 3 State Key Laboratory of Crop Biology, Shandong Key Laboratory of Crop Biology, College of Life Sciences, Shandong Agricultural University, Tai’an, 271018 P. R. China; 4 School of Agriculture, Ludong University, Yantai, 264025 P. R. China

**Keywords:** Chlorophyll fluorescence, Chloroplast ultrastructure, Modulated 820 nm reflection, Oxidative stress, Photoinhibition

## Abstract

**Background:**

*Glycine soja* is a halophytic soybean native to saline soil in Yellow River Delta, China. Photosystem I (PSI) performance and the interaction between photosystem II (PSII) and PSI remain unclear in *Glycine soja* under salt stress. This study aimed to explore salt adaptability in *Glycine soja* in terms of photosystems coordination.

**Results:**

Potted *Glycine soja* was exposed to 300 mM NaCl for 9 days with a cultivated soybean, *Glycine max*, as control. Under salt stress, the maximal photochemical efficiency of PSII (Fv/Fm) and PSI (△MR/MR_0_) were significantly decreased with the loss of PSI and PSII reaction center proteins in *Glycine max*, and greater PSI vulnerability was suggested by earlier decrease in △MR/MR_0_ than Fv/Fm and depressed PSI oxidation in modulated 820 nm reflection transients. Inversely, PSI stability was defined in *Glycine soja*, as △MR/MR_0_ and PSI reaction center protein abundance were not affected by salt stress. Consistently, chloroplast ultrastructure and leaf lipid peroxidation were not affected in *Glycine soja* under salt stress. Inhibition on electron flow at PSII acceptor side helped protect PSI by restricting electron flow to PSI and seemed as a positive response in *Glycine soja* due to its rapid recovery after salt stress. Reciprocally, PSI stability aided in preventing PSII photoinhibition, as the simulated feedback inhibition by PSI inactivation induced great decrease in Fv/Fm under salt stress. In contrast, PSI inactivation elevated PSII excitation pressure through inhibition on PSII acceptor side and accelerated PSII photoinhibition in *Glycine max*, according to the positive and negative correlation of △MR/MR_0_ with efficiency that an electron moves beyond primary quinone and PSII excitation pressure respectively.

**Conclusion:**

Therefore, photosystems coordination depending on PSI stability and rapid response of PSII acceptor side contributed to defending salt-induced oxidative stress on photosynthetic apparatus in *Glycine soja*. Photosystems interaction should be considered as one of the salt adaptable mechanisms in this halophytic soybean.

## Background

It is a great challenge to supply increasing population with enough food in future under the background of worldwide land degradation [1, 2]. Soil salinization is a major kind of land degradation and poses a serious threat to sustainable agricultural production. Irrigated farmland usually confronts secondary salinization because of unreasonable irrigation and fertilization, whereas large areas of saline land due to primary salinization are distributed in coastal zone and inland arid region [1]. In contrast to single soil improvement, biosaline agriculture has been proposed as an environmental friendly approach for managing saline land [3–5]. Halophytic crops are important germplasm resource, and besides direct planting in saline land, they also can be used for breeding new genotypes with salt tolerance by traditional hybridization or gene transformation. However, it is better to ascertain physiological mechanisms for adapting to saline stress in halophytes beforehand.

Salt stress disturbs plant metabolisms and inhibits plant growth by inducing osmotic stress and ionic toxicity, and as a salt-induced secondary stress, oxidative damage on biological macromolecules often arises [6–8]. Correspondingly, plants have evolved some defensive mechanisms such as root Na^+^ exclusion, osmolyte synthesis and antioxidant induction. These defensive mechanisms generally work more effectively in halophytes, and additionally, some special defensive behaviors exist in halophytes for their survival in saline land, such as salt secretion by glands and salt accumulation in vacuoles as osmolytes [8–12]. Plant survival and growth largely depend on photosynthesis. Photosynthesis is very sensitive to salt stress, and photosynthetic capacity seems to be a feasible criterion for differentiating plant salt tolerance [13–18]. In general, stomatal limitation on photosynthesis initially occurs due to salt-induced osmotic stress, and the inhibition on dark enzymatic processes can further reduce CO_2_ fixation [17, 19, 20]. As a consequence, excitation pressure in chloroplast may be elevated to induce photosystems photoinhibition with excess ROS production [21, 22]. At present, most studies focus on salt-induced photosystem II (PSII) photoinhibition, and halophytes generally have higher PSII photochemical capacity and CO_2_ assimilation rate than the glycophytic relatives under salt stress [14, 23, 24]. However, it remains unclear whether PSII components involving reaction center, donor and acceptor electron carriers have the uniform response in halophyte under salt stress.

Up to now, very limited attention has been paid to photosystem I (PSI) under salt stress, let alone the interaction between PSII and PSI [16]. In our recent studies, PSI was proved to be a crucial photoinhibition site in some glycophytic crops under salt stress [18, 25, 26]. Unlike PSII, it is hard to repair damaged PSI [22]. PSI photoinhibition can intensify PSII excitation pressure through feedback inhibition on electron transport and aggravate PSII photoinhibition [27, 28]. Accordingly, PSI inactivation has been found in susceptible plant species or cultivars with weak adaptability to abiotic stresses [18, 25, 28]. In contrast, PSII photoinhibition which restricts electron donation to PSI can help to prevent PSI photoinhibition by reducing ROS generation through Mehler reaction at PSI acceptor side [29–33]. Therefore, PSII and PSI coordination plays an important role in protecting the whole photosynthetic apparatus. In addition to great ability to defense ionic toxicity and osmotic stress, halophytes can effectively dissipate excitation energy in chloroplast and scavenge ROS for protecting PSII from oxidative damage, and PSII photoinhbition is rarely reported in halophytes under salt stress [14, 23, 24, 34]. Notably, PSII stability can elevate the possibility of PSI oxidative injury in halophytes particularly under long-term severe salt stress with tremendous decrease in CO_2_ assimilation. Up to now, PSII and PSI coordination has not been reported in halophytes upon salt stress, and it remains unknown whether halophytes can protect PSI against photoinhibition by the flexible response of PSII.

Wild soybeans are precious germplasm resources for improving environmental adaptability in cultivated soybean. *Glycine cyrtoloba* is a wild soybean species native to saline soil in Australian beach, and a series of studies have demonstrated its high salt tolerance in terms of inhibiting Na^+^ accumulation, photosynthetic activity, antioxidant activity, cyclic electron flow around PSI and excitation energy dissipation [23, 34–36]. In China, a halophytic soybean, *Glycine soja*, grows in coastal saline land in Yellow River Delta, and similar to *Glycine cyrtoloba*, *Glycine soja* also can effectively retard toxic ions accumulation and maintain high photosynthetic activity under salt stress [24, 37, 38]. In a recent study, we systematically illustrated salt tolerance in *Glycine soja* from the aspects of root ions flux, antioxidant system, osmotic regulation and photosynthesis [14]. Nonetheless, photosynthetic analysis was mainly concentrated on gas exchange characterization in these halophytic soybeans upon salt stress, and PSII salt tolerance was only defined by no obvious change in the maximal photochemical efficiency of PSII (Fv/Fm). Fv/Fm cannot reflect heterogeneous behaviors of PSII components [39], and PSI performance and the coordination between PSII and PSI remain unclear in the halophytic soybean under salt stress. In this study, we attached importance to photosystem performance and photosynthetic electron transport, and aimed to deeply reveal salt adaptability in *Glycine soja* by elucidating photosystems coordination. This study can provide an insight to crop salt tolerance and may assist in soybean germplasm improvement.

## Results

### Gas exchange, electron transport rate and PSII excitation pressure

**Fig. 1:**
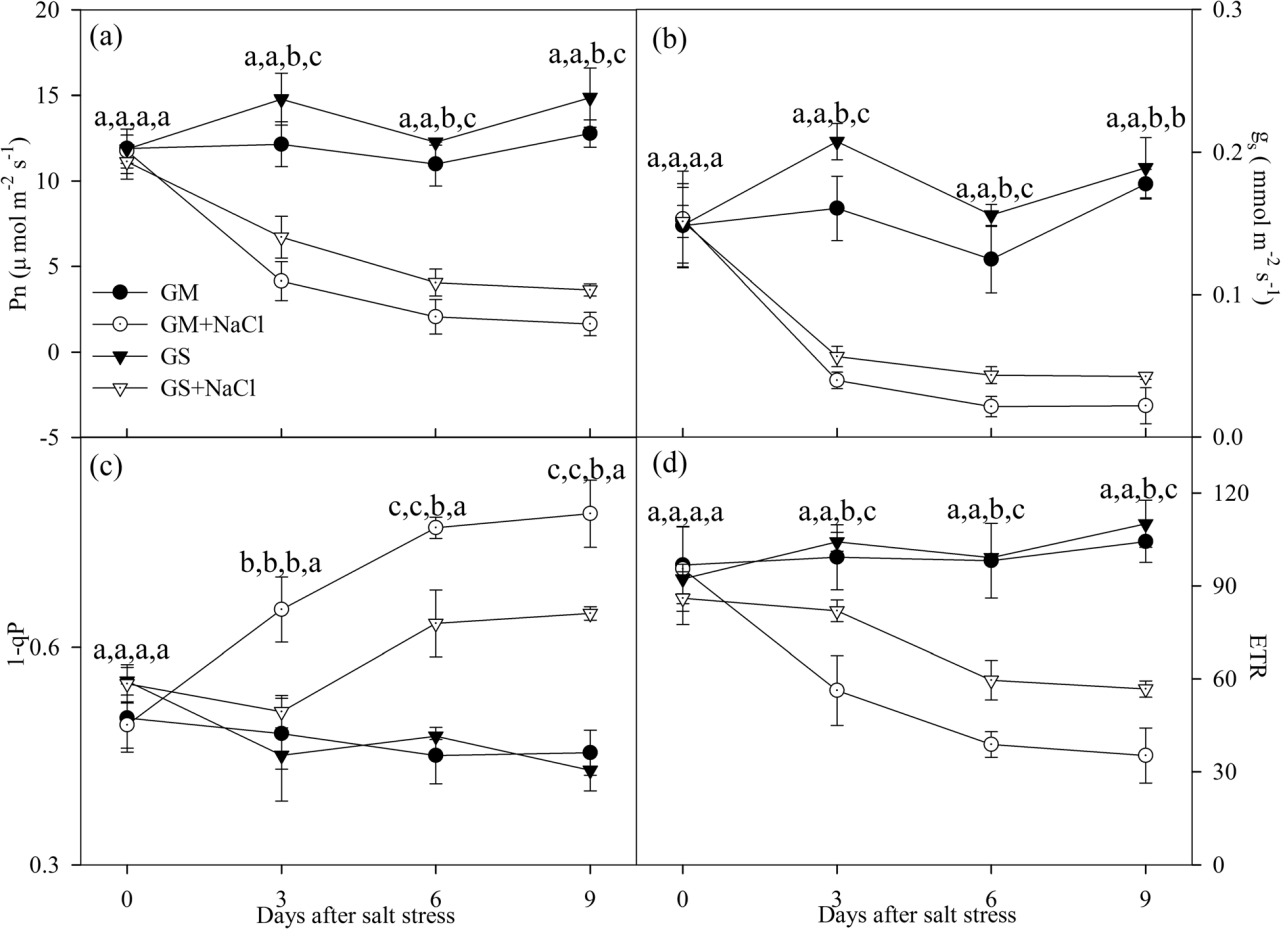
Photosynthetic rate (Pn, **a**), stomatal conductance (g_s_, **b**), PSII excitation pressure (1-qP, **c**) and PSII electron transport rate (ETR, **d**) in *Glycine max* (GM, circles) and *Glycine soja* (GS, triangles) exposed to 0 (closed symbols) and 300 mM (open symbols) NaCl. Data in the figure indicate the mean of five replicates (±SD). Different letters indicate significant difference among GS, GM, GS + NaCl and GM + NaCl at *P* < 0.05

Photosynthetic rate (Pn), stomatal conductance (g_s_) and PSII electron transport rate (ETR) were significantly decreased in the leaves of *Glycine soja* and *Glycine max* under salt stress, and greater decrease was noted in *Glycine max* (Fig. 1a, b, d). Under salt stress, PSII excitation pressure (1-qP) was significantly increased by 35.6% in *Glycine max* at day 3, and the increase reached 72.5% at day 9 (Fig. 1c). After 6 days of salt stress, significant increase in 1-qP was observed in *Glycine soja*, and the increase was up to 50.3% at day 9 (Fig. 1c).

### Prompt chlorophyll *a* fluorescence (PF), modulated 820 nm reflection transients (MR) and delayed chlorophyll *a* fluorescence (DF)

**Fig. 2:**
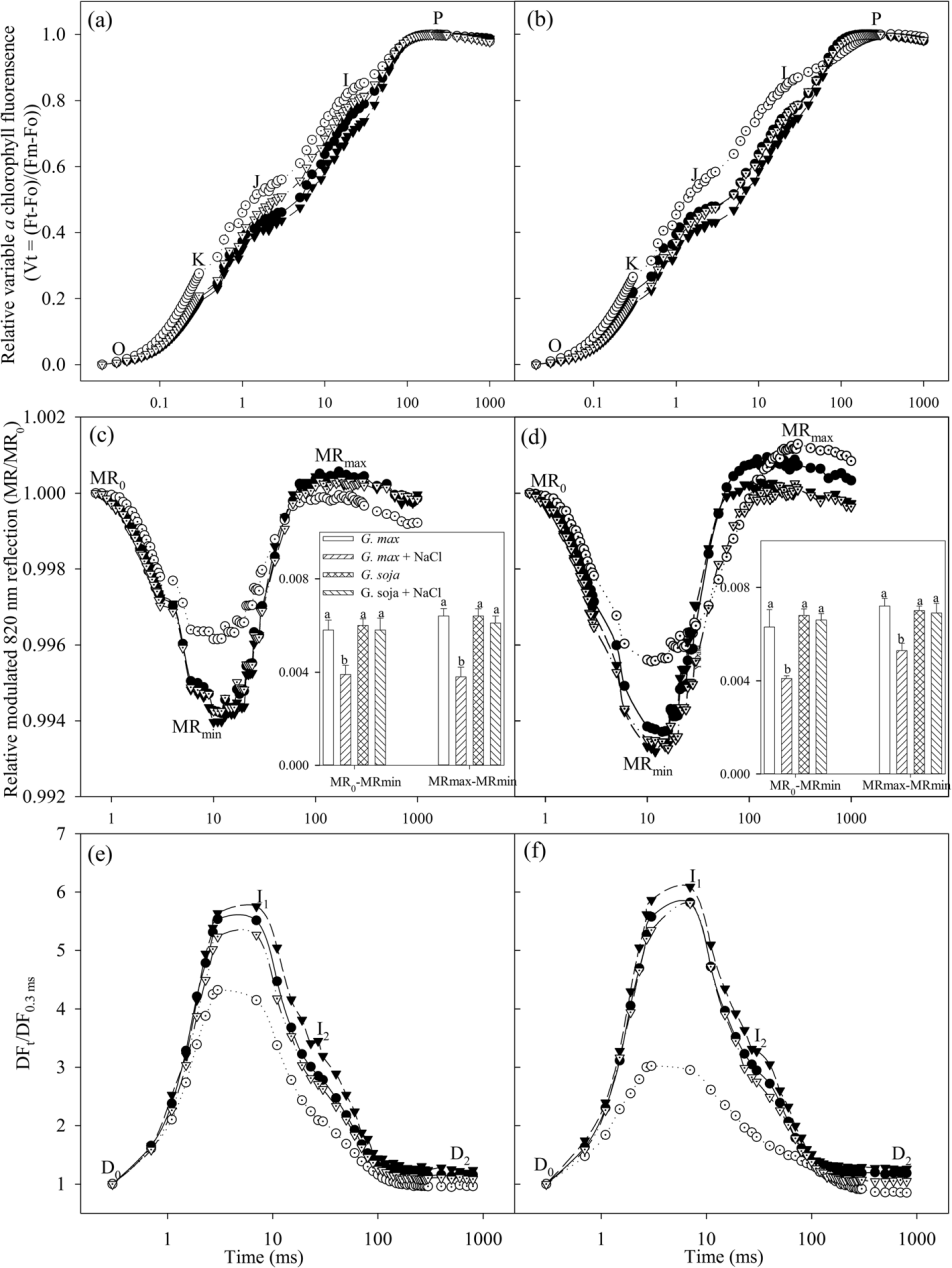
Transients of prompt chlorophyll a fluorescence **a**, **b**, modulated 820 nm reflection **c**, **d**, and delayed chlorophyll a fluorescence **e**, **f** in *Glycine max* (circles) and *Glycine soja* (triangles) exposed to 0 (closed symbols) and 300 mM (open symbols) NaCl for 3 (left panels) and 9 (right panels) days. O, K, J, I and P indicate the specific steps in chlorophyll *a* fluorescence transient. MR_0_ is the value of modulated 820 nm reflection at the onset of red light illumination (0.7 ms, the first reliable MR measurement). MR_0_-MRmin and MRmax-MRmin were PSI oxidation and re-reduction amplitude, respectively. The data of MR_0_-MRmin and MRmax-MRmin indicate mean of five replicates (±SD), and different letters on error bars indicate significant difference at *P* < 0.05. Do, I1, I2, D2 indicate initial point, the first (7 ms) and second (50 ms) maximal peaks and minimum point in delayed chlorophyll *a* fluorescence curves. DF_0.3ms_ is the initial microsecond delayed fluorescence signal at 0.3 ms. The signals were plotted on a logarithmic time scale, and each curve is the average of five replicates

If the re-oxidation of primary quinone (Q_A_) and plastoquinone (PQ) are inhibited, J and I steps will appear [40, 41]. After 3 days of salt stress, J and I steps were obviously elevated in *Glycine max* (Fig. 2a), and the elevation of J and I steps became greater upon salt stress for 9 days (Fig. 2b), suggesting that PQ re-oxidation and electron transfer at PSII side beyond Q_A_ were inhibited. K step usually arises around 300 μs due to the injury on OEC at PSII donor side [42, 43]. After 3 days of salt stress, PSII donor side was impaired in *Glycine max* according to the appearance of K step (Fig. 2a). Comparatively, salt stress induced smaller elevation of J and I steps with no change in K step in *Glycine soja* (Fig. 2a, b).

MR signals are presented by MR/MR_0_ ratio, where MR_0_ is the value at onset of actinic illumination (at 0.7 ms). PSI oxidation was initiated with the decrease in MR/MR_0_ from MR_0_ to the minimal value (MRmin) in MR transient, and subsequently, the increase of MR/MR_0_ to the maximal level (MRmax) indicates PSI re-reduction. MR transient remarkably changed with significantly decreased MR_0_-MRmin and MRmax-MRmin in *Glycine max* under salt stress (Fig. 2c, d), suggesting that both PSI oxidation and re-reduction were negatively affected. In contrast, PSI oxidation and re-reduction were not inhibited by salt stress in *Glycine soja*, as no obvious change was found in MR_0_-MRmin, MRmax-MRmin and MR transient (Fig. 2c, d). Under salt stress, DF transient in *G. max* was prominently depressed with significant decrease in I_1_ and I_2_ peaks, but I_1_ and I_2_ peaks were slightly declined in *Glycine soja* (Fig. 2e, f).

### Immunoblot analysis, PSII performance and the maximal photochemical capacity of PSI

**Fig. 3:**
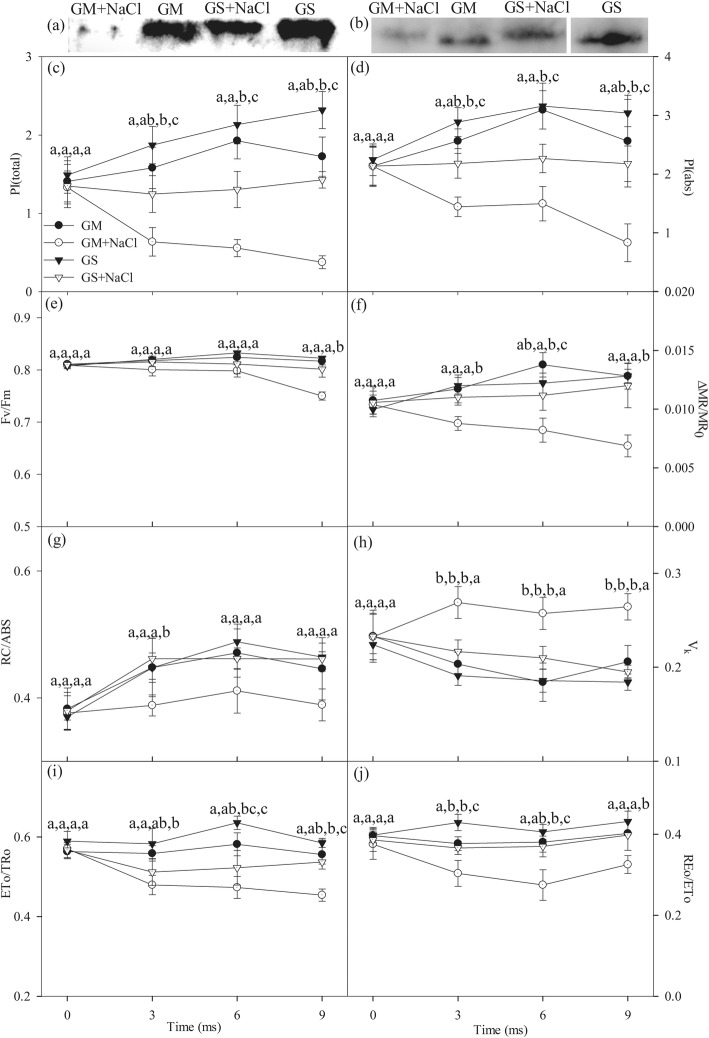
Immunoblot analysis of reaction center proteins of PSI (PsaA, **a**) and PSII (PsbA, **b**) in the leaves of *Glycine max* (GM) and *Glycine soja* (GS) after 9 days of salt stress with 300 mM NaCl. Total performance index (PI_total_, **c**), PSII performance index (PI_abs_, **d**), the maximal photochemical efficiency of PSII (Fv/Fm, **e**) and PSI (△MR/MR_0_, **f**), primary quinone reducing reaction centers per PSII antenna chlorophyll (RC/ABS, **g**), variable fluorescence intensity at K step (V_k_, **h**), probability that an electron moves beyond primary quinone (ETo/TRo, **i**) and probability with which an electron from the intersystem electron carriers is transferred to reduce end electron acceptors at the PSI acceptor side (REo/ETo, **j**) in the leaves of *Glycine max* (circles) and *Glycine soja* (triangles) exposed to 0 (closed symbols) and 300 mM (open symbols) NaCl. Data in the figure indicate the mean of five replicates (±SD). Different letters indicate significant difference among GS, GM, GS + NaCl and GM + NaCl at *P* < 0.05. The blot signals are cropped from the full-length blot images in Additional file 2: Figure S2 and Additional file 3: Figure S3

After 9 days of salt stress, PSI reaction center protein (PsaA) and PSII reaction center protein (PsbA) abundance were obviously declined in *Glycine max* rather than *Glycine soja* (Fig. 3a, b). Salt stress significantly declined total performance index (PI_total_) and PSII performance index (PI_abs_) in *Glycine max* and *Glycine soja*, and greater decrease was noted in *Glycine max* (Fig. 3c, d). Similarly, salt stress also induced greater decrease in probability with which an electron moves beyond Q_A_ (ETo/TRo) and from the intersystem electron carriers to reduce PSI end electron acceptors (REo/ETo) in *Glycine max* than *Glycine soja* (Fig. 3i, j). Significant decrease in the maximal photochemical capacity of PSI (△MR/MR_0_) was noted in *Glycine max* rather than *Glycine soja* under salt stress, and salt-induced decrease in Q_A_ reducing reaction centers per PSII antenna chlorophyll (RC/ABS) was also observed in *Glycine max* (Fig. 3f, g). In line with elevated K step (Fig. 2a, b), salt-induced significant increase in variable fluorescence intensity at K step (V_k_) was also found in *Glycine max* (Fig. 3h). When salt stress was prolonged to 9 days, Fv/Fm was significantly decreased in *Glycine max*, but the decrease in Fv/Fm was not significant in *Glycine soja* (Fig. 3e).

### Recovery of photosystem performance

One day after the cease of salt stress, PI_total_, PI_abs_, ETo/TRo and REo/ETo in salt-treated *Glycine soja* rapidly recovered to the normal level in *Glycine soja* without salt treatment, implying the high salt tolerance of photosystems in *Glycine soja* (Table 1). Probably due to irreversible damage on photosystems, these parameters did not show recovery in *Glycine max* despite the cease of salt stress (Table 1).

**Table 1:** Recovery of Total performance index (PI_total_), PSII performance index (PI_abs_), efficiency that an electron moves beyond primary quinone (ETo/TRo,) and probability with which an electron from the intersystem electron carriers is transferred to reduce end electron acceptors at the PSI acceptor side (REo/ETo) for one day in the leaves of *Glycine soja* (GS) and *Glycine max* (GM) after 9 days of salt stress with 300 mM NaCl. Data in the table indicate the mean of five replicates (±SD). Within each row, means followed by the same letters are not significantly different between salt treatment and control at *P* < 0.05

Parameters	GM	GM + NaCl	GS	GS + NaCl
PI_total_	1.82 ± 0.33a	0.58 ± 0.21b	2.11 ± 0.19a	2.03 ± 0.13a
PI_abs_	2.64 ± 0.48a	0.78 ± 0.12b	2.87 ± 0.58a	2.69 ± 0.41a
ETo/TRo	0.54 ± 0.02a	0.43 ± 0.03b	0.56 ± 0.02a	0.54 ± 0.04a
REo/ETo	0.41 ± 0.03a	0.32 ± 0.01b	0.42 ± 0.03a	0.41 ± 0.01a

### The coordination between PSI and PSII

**Fig. 4:**
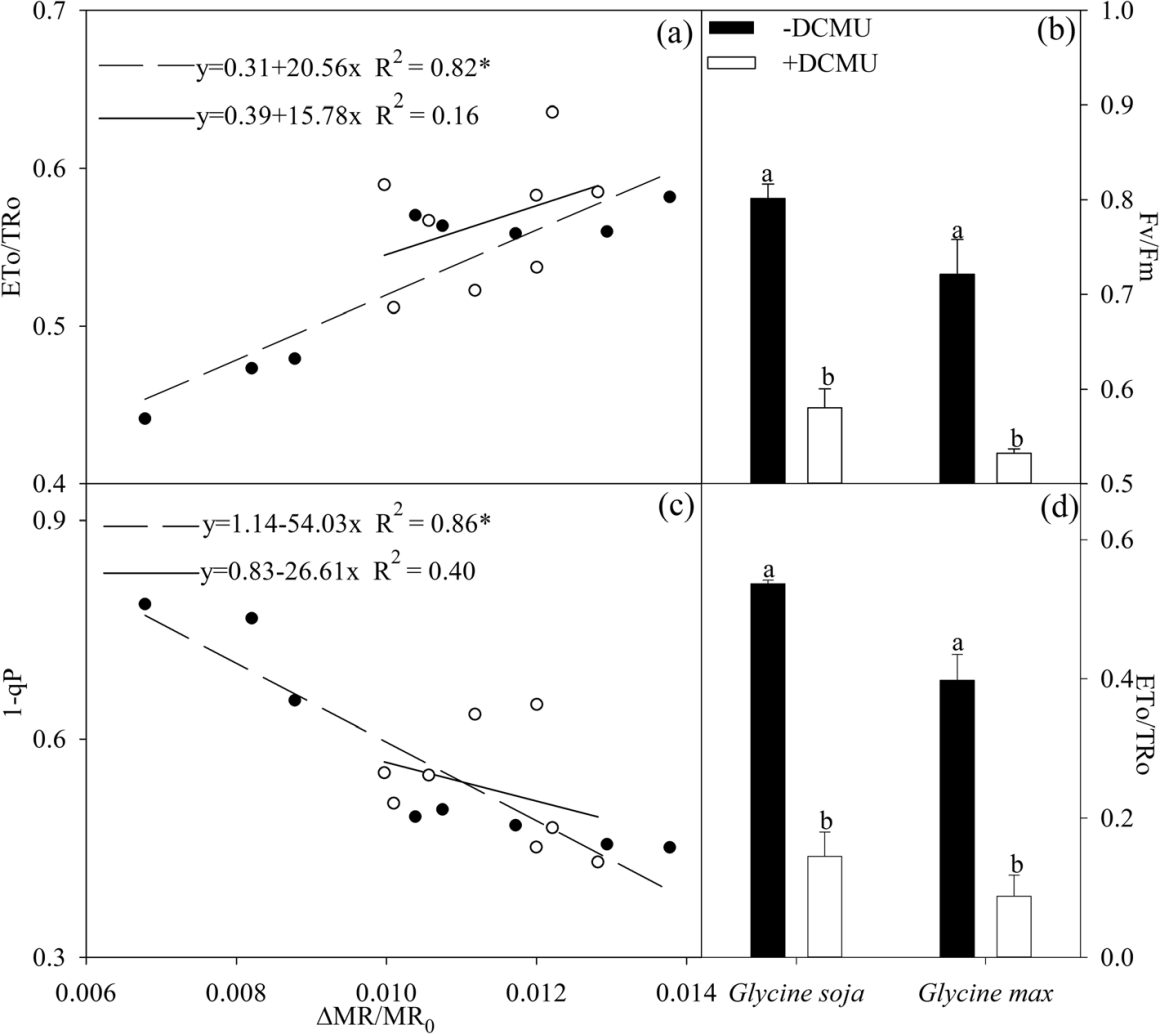
Regression analysis of the maximal photochemical efficiency of PSII (△MR/MR_0_) with probability that an electron moves beyond primary quinone (ETo/TRo, **a**) and PSII excitation pressure (1-qP, **c**) in *Glycine max* (closed symbols) and *Glycine soja* (open symbols). * Indicates that the correlation was significant at *P* < 0.01. The maximal photochemical efficiency of PSII (Fv/Fm, **b**) and ETo/TRo (**d**) in *Glycine max* and *Glycine soja* in the absence or presence of 3-(3,4-dichlorfenyl)-1,1-dimethylkarbonyldi-amid (DCMU) after 9 days of salt stress with 300 mM NaCl. For reagent treatment, the leaves after 6 days of salt stress with 300 mM NaCl were immersed in 0 or 70 μM DCMU for 3 h in the dark. The data of Fv/Fm (**b**) and ETo/TRo (**d**) indicate the mean of five replicates (±SD), and different letters on error bars indicate significant difference between DCMU treatment and control at *P* < 0.05

△MR/MR_0_ was significantly and positively correlated with ETo/TRo in *Glycine max* under salt stress, while significant and negative correlation was noted between △MR/MR_0_ and 1-qP (Fig. 4a, c). However, there was very low correlation of △MR/MR_0_ with ETo/TRo and 1-qP in *Glycine soja* (Fig. 4a, c). The application of DCMU, an inhibitor blocking the electron transport from Q_A_^−^ to Q_B_^−^, caused much greater decrease in Fv/Fm and ETo/TRo in *Glycine max* and *Glycine soja* after 9 days of salt stress (Fig. 4b, d).

### Fv/Fm with presence of chloramphenicol

**Fig. 5:**
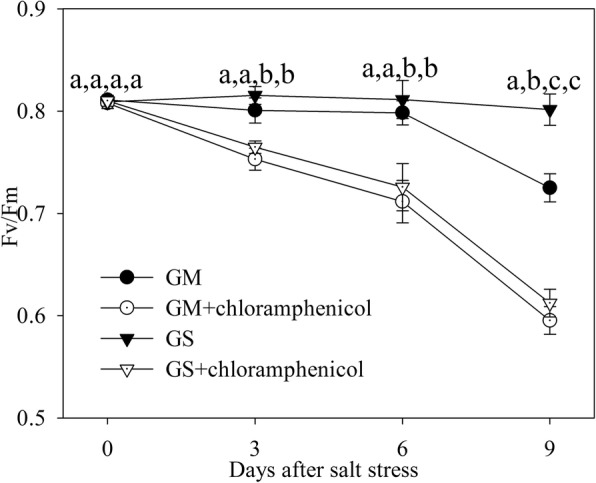
The maximal photochemical efficiency of PSII (Fv/Fm) in *Glycine max* (circles) and *Glycine soja* (triangles) exposed to 300 mM NaCl in the absence (closed symbols) or presence (open symbols) of chloramphenicol. For reagent treatment, leaves of *Glycine max* and *Glycine soja* were immersed in 0 or 1 mM chloramphenicol for 3 h in the dark before salt stress. Data in the figure indicate the mean of five replicates (±SD). Different letters indicate significant difference among GS, GM, GS + chloramphenicol and GM + chloramphenicol at *P* < 0.05

Chloramphenicol, an inhibitor of D1 protein de novo synthesis, was used to clarify whether the difference of D1 protein de novo synthesis was responsible for unequal PSII photoinhibition in *Glycine max* and *Glycine soja*. Due to the application of chloramphenicol, Fv/Fm was greater decreased to a similar level in *Glycine max* and *Glycine soja* upon salt stress (Fig. 5), suggesting that the negative effects of salt stress was imposed on PSII mainly by impeding D1 protein repair.

### Lipid peroxidation and H_2_O_2_ content

**Fig. 6:**
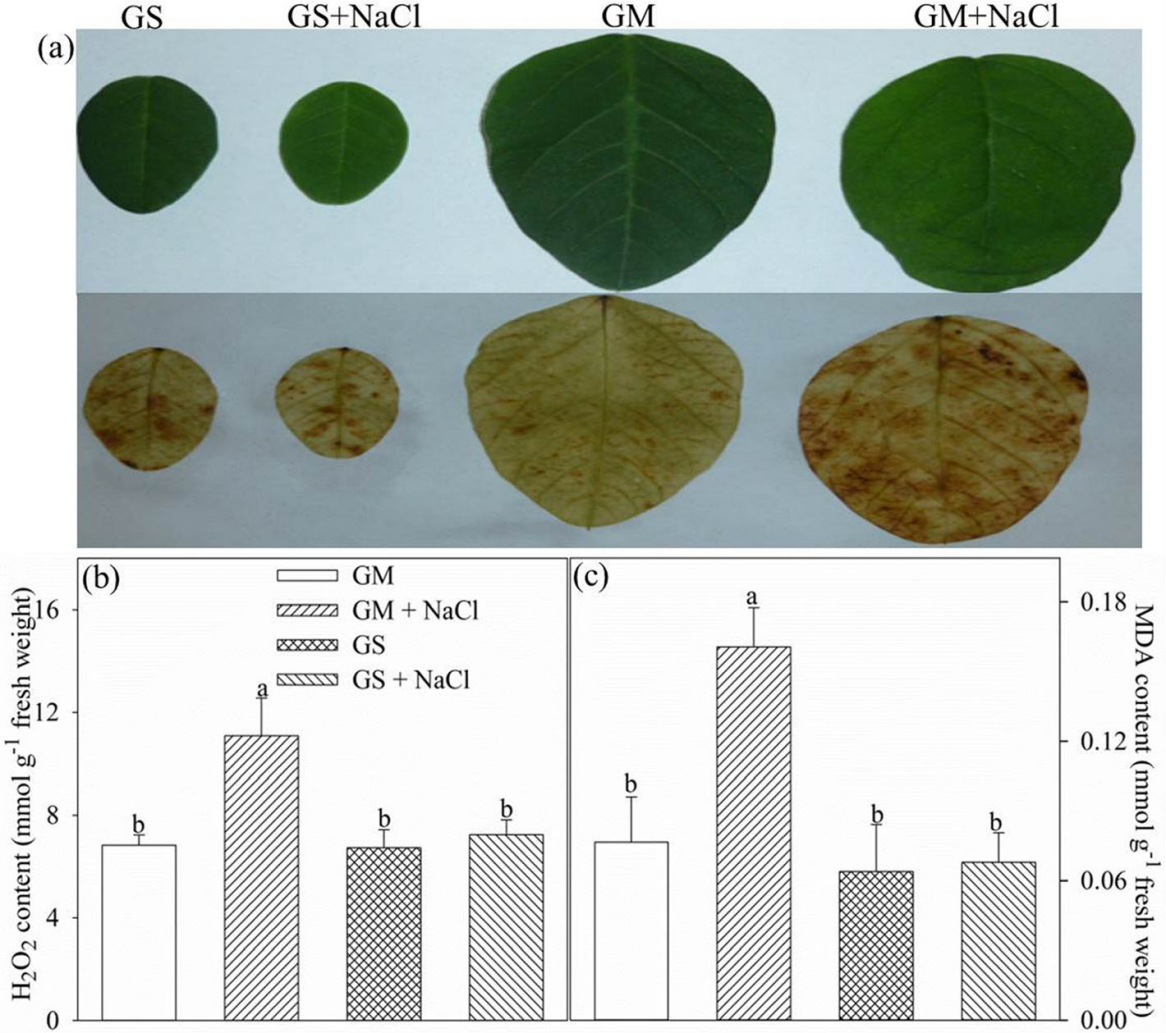
Histochemical detection of H_2_O_2_ (**a**), malondialdehyde (MDA, **b**) and H_2_O_2_ (c**)** contents in the leaves of *Glycine max* (GM) and *Glycine soja* (GS) after 9 days of salt stress with 300 mM NaCl. Data in the figure indicate the mean of five replicates (±SD), and different letters on error bars indicate significant difference between salt treatment and control at *P* < 0.05

Malondialdehyde (MDA) content reflects the level of lipid peroxidation in plant tissue. After 9 days of salt stress, MDA and H_2_O_2_ content was significantly increased in the leaves of *Glycine max* in contrast to no remarkable change in the leaves of *Glycine soja*, and consistently, histochemical staining with 3,3-diaminobenzidine also suggested the obvious increase of H_2_O_2_ content in the leaves of *Glycine max* rather than *Glycine soja* (Fig. 6).

### Ultrastructure of leaf chloroplast

**Fig. 7:**
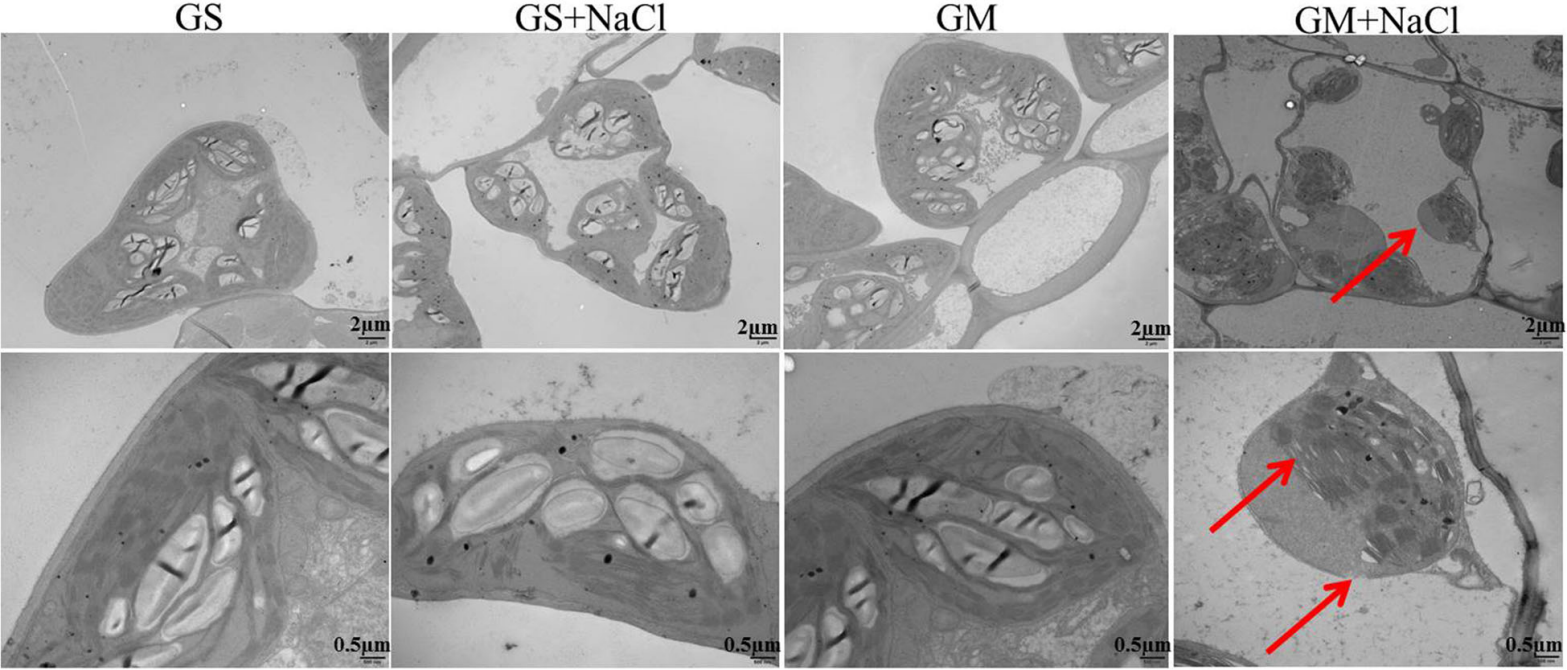
Ultrastructure of leaf chloroplast in *Glycine soja* (GS) and *Glycine max* (GM) after 9 days of salt stress with 300 mM NaCl. Red arrows indicated separated chloroplast from cell wall with great reduction of starch granules, disintegrated chloroplast envelope and distended thylakoids in *Glycine max* upon salt stress

After 9 days of salt stress, no obvious change occurred in mesophyll cell and chloroplast ultrastructure in *Glycine soja*, illustrating its high salt tolerance (Fig. 7). Nonetheless, chloroplast tended to separate from cell wall with great reduction of starch granules in *Glycine max* upon salt stress, and in addition to disintegrated chloroplast envelope, distended and loosen thylakoids were also detected (Fig. 7). The damage on chloroplast ultrastructure was accordant with salt-induced great depression on photosystems performance in *Glycine max*.

## Discussion

As with our previous study [14], tremendous decrease in Pn with stomatal closure was verified in *Glycine max* and *Glycine soja* under severe salt stress (Fig. 1a, b). The great depression on CO_2_ assimilation posed a big threat to photosynthetic apparatus by increasing the possibility of ROS generation through feedback inhibition on photosynthetic electron transport. Actually, elevated lipid peroxidation and H_2_O_2_ concentration confirmed salt-induced oxidative stress in the leaves of *Glycine max* (Fig. 6). Photosystems photoinhibition usually arises in parallel with elevated leaf lipid peroxidation under environmental stresses, and notably, the negative correlation of ROS production with PSI and PSII photochemical capacity under salt stress has been evidenced [18, 25, 28, 44]. As a result, unchanged lipid peroxidation and H_2_O_2_ concentration implied that photosystems were less endangered by salt-induced oxidative stress in *Glycine soja* than *Glycine max*.

Consistently, salt-induced PSII and PSI photoinhibition was noted in *Glycine max* rather than *Glycine soja* according to the variation of Fv/Fm and △MR/MR_0_, and less decrease in PI_total_ corroborated better performance of photosynthetic apparatus in *Glycine soja* (Fig. 3c, e, f). In particular, salt-induced destruction of chloroplast and thylakoid ultrastructure confirmed the serious depression on photosystems performance in *Glycine max* (Fig. 7). PSI vulnerability usually lies in sensitive plants and seems as a feasible criterion for discerning plant tolerance to abiotic stresses because of its threat to the entire photosynthetic apparatus [28]. In this study, PSI stability was defined in *Glycine soja* under salt stress due to unobvious change in △MR/MR_0_ and PsaA abundance (Fig. 3a, f). In contrast, PSI vulnerability was observed in *Glycine max*, indicated by salt-induced decrease in △MR/MR_0_ at day 3 before the occurrence of PSII photoinhibition (Fig. 3e, f). Therefore, high salt adaptability should include PSI stability in the halophytic soybean. PI_abs_ comprehensively reflects PSII performance and is more sensitive than Fv/Fm [45, 46]. After 3 days of salt stress, PSII performance was already depressed not only in *Glycine max* but also in *Glycine soja*, and less decease in PI_abs_ suggested greater PSII stability in *Glycine soja* (Fig. 3d). Under salt stress, PSII donor and acceptor sides were initially impaired with declined amount of active PSII reaction centers in *Glycine max* (Fig. 3g, i, j), and PSII reaction center was damaged later due to lowered Fv/Fm and PsbA abundance (Fig. 3b, e). Comparatively, only PSII acceptor side was influenced by salt stress in *Glycine soja*, and the influence was lighter than that in *Glycine max* in light of less decrease in ETo/TRo and smaller elevation of J step (Fig. 2a, b and 3i). Similar to previous study, PSII stability was verified in the halophytic soybean under salt stress [14, 24], however, the rapid response from PSII acceptor side was revealed in this study. Delayed chlorophyll *a* fluorescence in microsecond domain is mostly related to Z^+^Q_A_^−^ state of PS II [47]. The occurrence of I_1_ peak in DF transients mainly results from accumulation of S_3_Z^+^P680Q_A_^−^ state, which relates to active reaction centers and electron transfer capacity at both donor and acceptor sides of PSII [47, 48]. Under salt stress, large decrease in I_1_ corroborated the damage on PSII components including reaction center, donor and acceptor sides in *Glycine max*, whereas slight decrease in I_1_ was coincident with the mild inhibition on electron transport at PSII acceptor side in *Glycine soja* (Fig. 2e, f). Overall, PSI stability and rapid response of PSII acceptor side were illustrated in the halophytic soybean under salt stress.

As reported in previous studies, antioxidant enzymes were stimulated by salt stress to a higher level in *Glycine soja* than *Glycine max* [14]. Apart from the stronger antioxidant protection, photosystems coordination also played an important role in counteracting photosystems photoinhibtion in *Glycine soja* exposed to severe salt stress. PSI photoinhibition derives from oxidation of iron-sulfur protein by ROS generated through Mehler reaction at PSI acceptor side, and the electron flow from PSII is essential for PSI photoinhibition [22]. Upon salt-induced great decrease in CO_2_ assimilation, the rapid response of PSII acceptor side restricted electron flow to PSI in *Glycine soja* and could help protect against PSI inhibition by reducing ROS generation (Fig. 1d). The experiment with DCMU application, which simulated the suppression of electron transport at PSII acceptor side caused by PSI photoinhibition, demonstrated that PSI photoinhibition accelerated PSII photoinhibition (Fig. 4b, d). PSI activity bore no relation to PSII excitation pressure and electron transport at PSII acceptor side in *Glycine soja* according to the correlation analysis of △MR/MR_0_ with 1-qP and ETo/TRo (Fig. 4a, c). Therefore, PSI stability was conductive to preventing the occurrence of PSII photoinhibition by alleviating feedback inhibition on electron transport in *Glycine soja* under salt stress. Inversely, PSI vulnerability elevated PSII excitation pressure in *Glycine max* under salt stress by inducing over-reduction of PSII acceptor side, indicated by positive correlation between △MR/MR_0_ and ETo/TRo and negative correlation between △MR/MR_0_ and 1-qP (Figs. 1c and 4a, c), and eventually resulted in PSII photoinhibition (Fig. 3e). The difference of salt-induced PSII photoinhibition between *Glycine max* and *Glycine soja* originated from the repair of photodamaged PSII rather than direct photodamage to PSII, because Fv/Fm decreased to the same level in presence of chloramphenicol (Fig. 5). The declined PSI re-reduction amplitude in MR transients conformed to PSII inactivation in *Glycine max* (Fig. 2c, d), as PSI re-reduction process mainly depended on electron donation from PSII. In spite of great restriction on electron flow to PSI due to PSII inactivation, PSI oxidation amplitude in MR transient was still depressed (Fig. 2c, d), which verified greater damage on PSI than PSII. I_2_ phase in DF transients is related to the prolonged reopening of PSII reaction centers by electron transfer from reduced quinone to plastoquinone (PQ) before full reduction of PQ pool [43, 44, 49]. In agreement with elevated I step and decreased REo/ETo, the decrease in I_2_ also suggested that PQ re-oxidation was inhibited due to greater damage on PSI (Fig. 2e, f). Therefore, the passive PSII photoinhibition could not effectively defend oxidative injury of PSI in *Glycine max* under salt stress. Nonetheless, salt stress also elevated I step and induced decrease in REo/ETo and I_2_ in *Glycine soja* (Figs. 2a, b, e, f and 3j), and the declined PQ re-oxidation seemed contradictory to unchanged PSI activity. PQ is located in cytochrome b6f complex (cyt b6f) by which electron transport from PSII to PSI is bridged, and the soluble primary PSI acceptor, Ferredoxin, binds the stromal site of cyt b6f to trigger PSI cyclic electron flow [50]. PSI cyclic electron flow is an important photoprotective pathway in plants under abiotic stress due to the reduction of electron donation to PSI and production of ATP for repairing photodamaged PSII [51–54]. We supposed that the declined PQ re-oxidation resulted from enhanced binding of cyt b6f with Fd for promoting PSI cyclic electron flow in *Glycine soja* under salt stress and caused feedback inhibition on electron transport beyond Q_A_ at PSII acceptor side. This inference should be established in future study by exploring the relation between photosystem coordination and PSI cyclic electron flow. However, at least, rapid recovery of ETo/TRo and REo/ETo after salt stress supported that it was a positive response to limit electron transport between PSII and PSI in *Glycine soja* for protecting photosynthetic apparatus (Table 1).

## Conclusion

To summarize, photosystems coordination which was dependent on PSI stability and rapid response of PSII acceptor side contributed to defending salt-induced oxidative stress on photosynthetic apparatus in *Glycine soja*. This study can deepen the knowledge about salt tolerance mechanism in the halophyte.

## Methods

### Plant material and treatment

*Glycine soja* is a halophytic soybean native to coastal saline soil in Yellow River Delta, China, and the seeds of *Glycine soja* were carefully collected from wild plants near the Yellow River Delta coastal wetland ecological experimental station, Chinese Academy of Sciences (N37°45′, E118°58′). The collected seeds have been identified by Prof. Hualing Xu and deposited in Dongying academy of agricultural sciences. Experimental research on plants including collection of plant material in this study did not violate any guideline or local legislation. Special permissions and ethical approval were not required to collect and use this wild soybean. *Glycine max* is a major cultivated soybean in China, and the seeds of *Glycine max* were obtained by sexual hybridization with yudou8 and zhongzuo90052–76 as the parents. The seeds of *Glycine max* were provided by Shandong academy of agricultural sciences. The seeds of *Glycine soja* and *Glycine max* are shown in Additional file 1: Figure S1, and the seed specimens were, respectively, conserved in Dongying academy of agricultural sciences and Shandong academy of agricultural sciences without voucher number.

The protocols for seed germination and seedling culture have been reported in our previous study [14]. In July 7, 2017, the seeds of *Glycine max* were soaked in distilled water for 8 h, while the seeds of *Glycine soja* were soaked in concentrated sulfuric acid for 10 min to remove the hard shell over the seeds. The seeds were transferred to petri dishes and laid between two sheets of filter paper for germinating at 25 °C in the dark, and Hoagland nutrient solution (pH 5.7) was sprayed to keep the filter paper wet. The Hoagland nutrient solution contains calcium nitrate tetrahydrate (945 mg L^− 1^), disodium EDTA (37 mg L^− 1^), potassium nitrate (506 mg L^− 1^), ferrous sulfate heptahydrate (28 mg L^− 1^), ammonium nitrate (80 mg L^− 1^), boric acid (6.2 mg L^− 1^), potassium iodide (0.83 mg L^− 1^), manganese sulfate (22 mg L^− 1^), potassium dihydrogen phosphate (136 mg L^− 1^), zinc sulfate (8.6 mg L^− 1^), magnesium sulphate (493 mg L^− 1^), sodium molybdate (0.25 mg L^− 1^), copper sulphate (0.025 mg L^− 1^) and cobalt chloride (0.025 mg L^− 1^). After germination, the seedlings were cultured with the same method reported in our previous study [14]. One month later, salt treatment was conducted on uniform plants by adding NaCl to Hoagland nutrient solution (pH, 5.7). NaCl concentration in Hoagland nutrient solution (pH, 5.7) was gradually elevated by 50 mM every day to reach the final treatment concentration (300 mM NaCl). The newest fully expanded leaves were sampled for measuring physiological and biochemical parameters. After 9 days of salt stress, NaCl in the culture medium were leached completely with Hoagland nutrient solution for examining the recovery of photosystem performance, as there was a small hole in the bottom of each pot for leaching salt.

### Measurements of gas exchange and modulated chlorophyll fluorescence

Gas exchange and modulated chlorophyll fluorescence parameters were simultaneously detected by using an open photosynthetic system (LI-6400XTR, Li-Cor, Lincoln, NE, USA) equipped with a fluorescence leaf chamber (6400–40 LCF, Li-Cor). The temperature and CO_2_ concentration were respectively set at 25 °C and 400 μmol mol^− 1^ in the leaf cuvette. Pn and g_s_ were simultaneously recorded. Modulated chlorophyll fluorescence was also recorded for calculating ETR and 1-qP according to previous studies [25, 55].

### Measurements of prompt chlorophyll fluorescence, modulated 820 nm reflection and delayed chlorophyll fluorescence transients

By using a multifunctional plant efficiency analyzer (MPEA, Hansatech, UK), PF, DF and MR transients were simultaneously recorded in the first 1 s illumination with red light, and MR signals were still detected in the following 10 s far red illumination. The redox state of PSI reaction center under continuous light can be detected by monitoring 820 nm reflection [40]. ΔMR/MR_0_, Fv/Fm, V_k_, RC/ABS, ETo/TRo, REo/ETo, PI_abs_ and PI_total_ were calculated according to previous studies [40, 56, 57].

All redox reactions of the photosynthetic electron transport are reversible, and the back electron transfer and charge recombination in PSII reaction center lead to delayed fluorescence emission from repopulated excited chlorophyll [47]. DF signals are recorded in dark intervals for excluding PF interference under the light [47, 57]. In this study, DF signals in microsecond domain were collected at 20 μs after turning off actinic light for constructing DF transients.

### Measurements of MDA and H_2_O_2_ contents and histochemical detection of H_2_O_2_

MDA content was measured by thiobarbituric acid reaction method for indicating lipid peroxidation degree [58]. Leaf tissues (0.5 g) were ground under liquid nitrogen and homogenized in 5 mL 0.1% TCA. The homogenate was centrifuged at 10000×*g* and 4 °C for 10 min to collect the supernatant for measuring MDA and H_2_O_2_ contents [25]. Leaves were vacuum-infiltrated with 0.1 mg ml^− 1^ 3, 3-diaminobenzidine in 50 mM tris-acetate solution (pH, 3.8) and incubated at room temperature in the dark for 24 h. Thereafter, the leaves were decolorized by immersion in boiling ethanol (80%) for 10 min and photographed [59].

### Isolation of thylakoid membranes and western blot

As with the method of Yan et al. [28], western blots of PsbA and PsaA were carried out through the procedures including protein extraction, SDS-PAGE gel, protein transfer to polyvinylidene fluoride membranes, incubation with specific antibodies and signal detection by chemiluminescence.

### Observation of chloroplast ultrastructure

Similar to Oustric et al. [60] with small modification, leaf pieces (1 mm^2^) were sampled, fixed in 2.5% glutaraldehyde in 100 mM phosphate buffer (pH, 7.2) for 2 h at room temperature and washed with the same buffer. The samples were post-fixed in 1% osmic acid in 100 mM phosphate buffer (pH, 7.2) at room temperature for 4 h, parched through a graded ethanol series (50–100%) and embedded in Spurr’s epoxy resin. An ultramicrotome (Leica ultracut R, Germany) was used to obtain ultra-sections (70 nm), and then, chloroplast ultrastructure was detected by a transmission electron microscope (JEM-1230, Japan) after staining these ultra-sections with uranyl acetate and lead phosphate.

### Statistical analysis

One-way ANOVA was carried out by using SPSS 16.0 (SPSS Inc., Chicago, IL, USA) for all sets of data. The values presented are the means of measurements with five replicate plants, and comparisons of means were determined through LSD test. Difference was considered significant at *P* < 0.05.

## Supplementary information

**Additional file 1 : Figure S1**. *Glycine soja* and *Glycine max* seeds, transients of prompt chlorophyll *a* fluorescence in *Glycine soja* and *Glycine max* before salt treatment, and the growth of *Glycine soja* and *Glycine max* in an artificial climatic chamber.

**Additional file 2 : Figure S2.** The original image of western blot of reaction center proteins of PSII (PsbA) protien in *Glycine max* (GM) and *Glycine soja* (GS) under salt stress. A red arrow indicates the cropped signals which were used in Fig. 3b.

**Additional file 3 : Figure S3.** The original image of western blot of reaction center proteins of PSI (PsaA) protien in *Glycine max* (GM) and *Glycine soja* (GS) under salt stress. A red arrow indicates the cropped signals which were used in Fig. 3a.

## Data Availability

The datasets used and/or analysed during the current study are available from the corresponding author on reasonable request.

## Abbreviations

Cyt b6f: Cytochrome b6f complex
ETo/TRo: Probability with which an electron moves beyond primary quinone
ETR: PSII electron transport rate
Fv/Fm: The maximal quantum yield of PSII
g_s_: Stomatal conductance
MDA: Malondialdehyde
Pn: Photosynthetic rate
PI_abs_: PSII performance index
PI_total_: Total performance index
PSI: Photosystem I
PSII: Photosystem II
RC/ABS: Primary quinone reducing reaction centers per PSII antenna chlorophyll
PQ: Plastoquinone
REo/ETo: Probability with which an electron from the intersystem electron carriers is transferred to reduce end electron acceptors at the PSI acceptor side
Q_A_: Primary quinone
ROS: Reactive oxygen species
V_k_: Variable fluorescence intensity at K step
△MR/MR_0_: The maximal photochemical capacity of PSI
ΦPSII: Actual photochemical efficiency of PSII
1-qP: Excitation pressure of PSII
