# Supplementary figures and images for: Salt adaptability in a halophytic soybean (Glycine soja) involves photosystems coordination
Source: BMC Plant Biol. 2020 Apr 10;20:155. doi: 10.1186/s12870-020-02371-x (PMC7149873; doi:10.1186/s12870-020-02371-x)

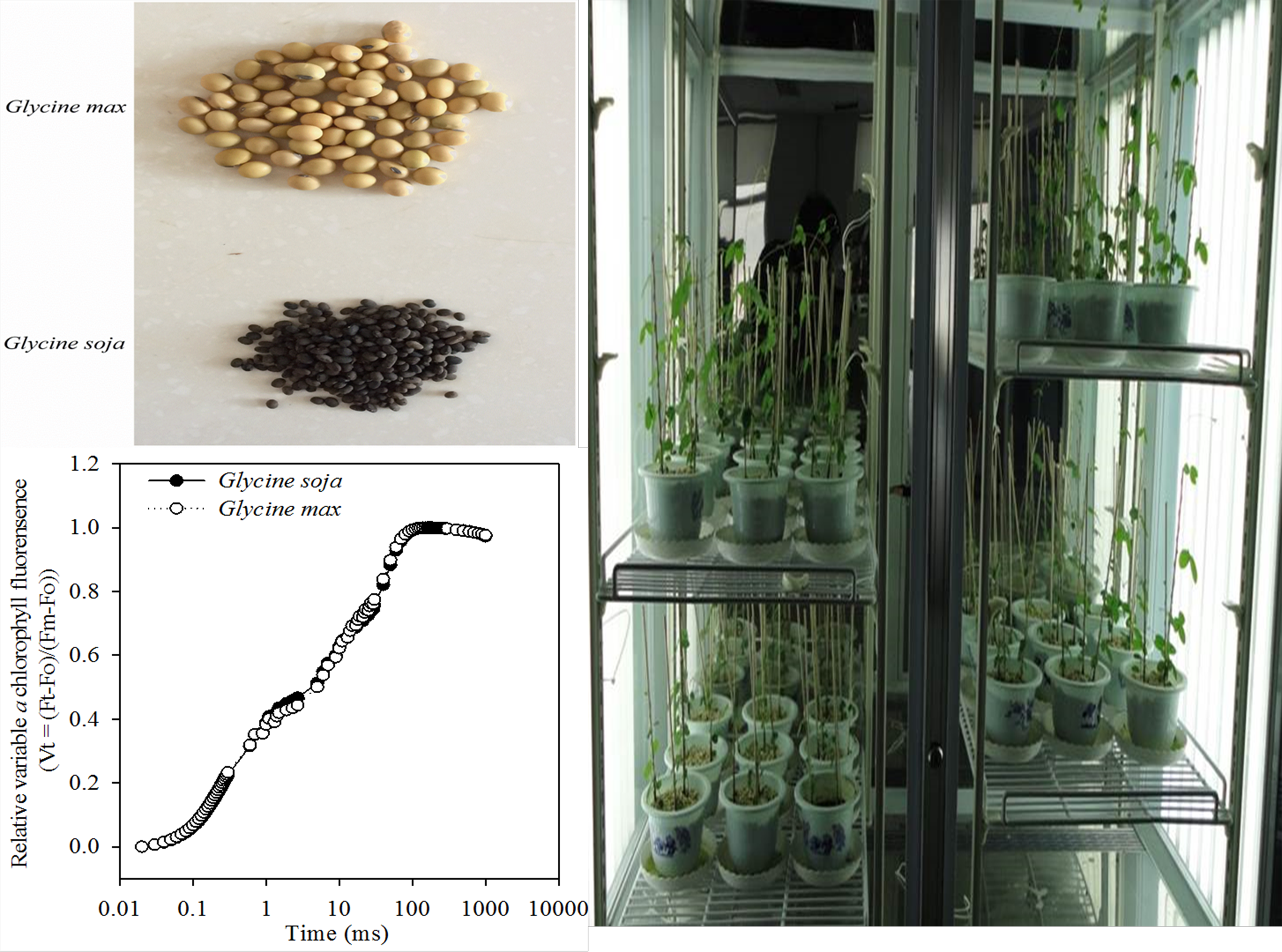

Supplement: Supplementary file 1 — Additional file 1 : Figure S1. Glycine soja and Glycine max seeds, transients of prompt chlorophyll a fluorescence in Glycine soja and Glycine max before salt treatment, and the growth of Glycine soja and Glycine max in an artificial climatic chamber. [file 12870_2020_2371_MOESM1_ESM.tif]

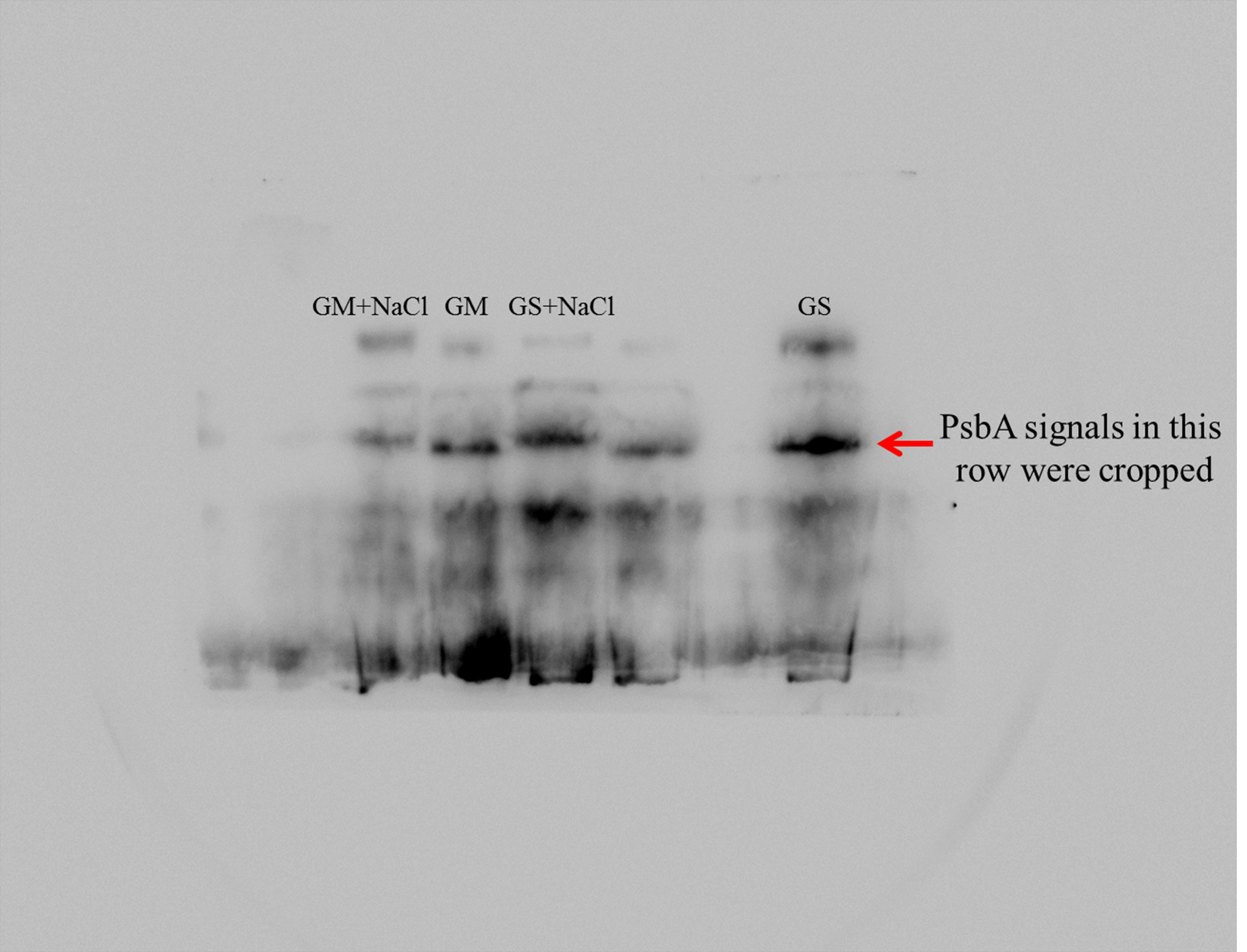

Supplement: Supplementary file 2 — Additional file 2 : Figure S2. The original image of western blot of reaction center proteins of PSII (PsbA) protien in Glycine max (GM) and Glycine soja (GS) under salt stress. A red arrow indicates the cropped signals which were used in Fig. 3b. [file 12870_2020_2371_MOESM2_ESM.tif]

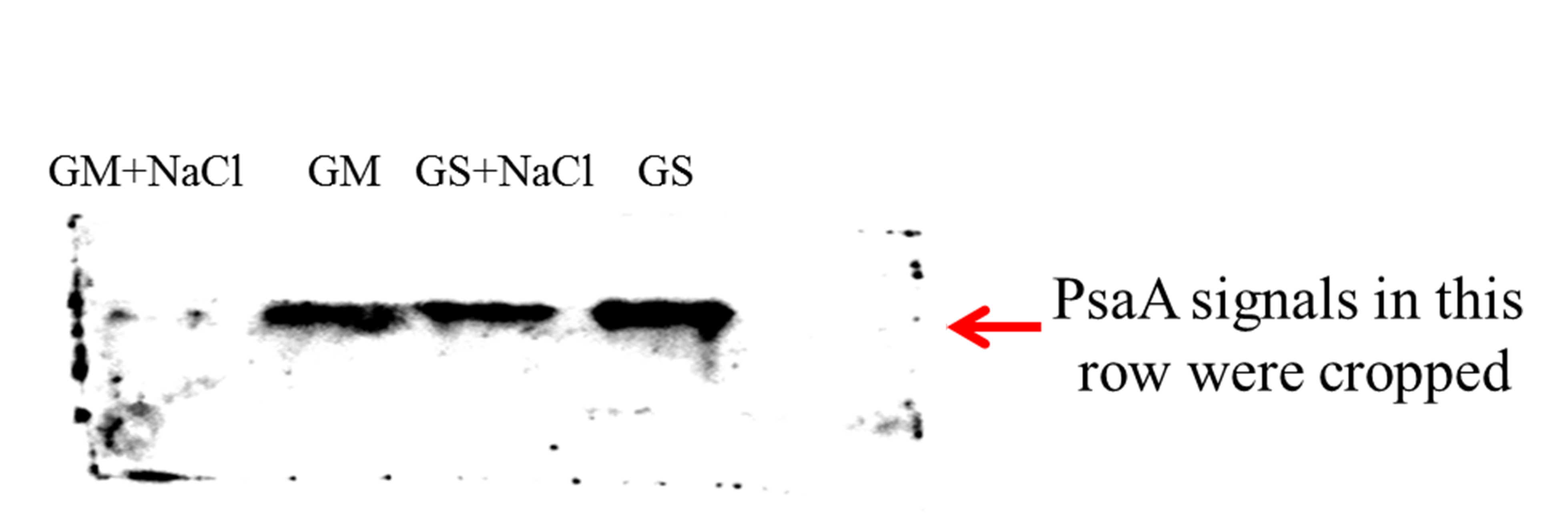

Supplement: Supplementary file 3 — Additional file 3 : Figure S3. The original image of western blot of reaction center proteins of PSI (PsaA) protien in Glycine max (GM) and Glycine soja (GS) under salt stress. A red arrow indicates the cropped signals which were used in Fig. 3a. [file 12870_2020_2371_MOESM3_ESM.tif]
